# Painless Concurrent Suprascapular and Axillary Neuropathy in a Young Elite Volleyball Player: A Case Report

**DOI:** 10.3390/diagnostics16172820

**Published:** 2026-09-02

**Authors:** Jaesung Yoo, Jae-Wook Park

**Affiliations:** Department of Orthopedic Surgery, Dankook University Hospital, 201 MangHyang-ro, Dongnam-gu, Cheonan 31116, Republic of Korea

**Keywords:** axillary nerve, peripheral nerve injuries, shoulder, suprascapular nerve, volleyball, electrodiagnosis, overhead athlete

## Abstract

**Background and Objectives**: Peripheral nerve injuries around the shoulder are increasingly recognized in athletes who perform repetitive overhead motions. Suprascapular neuropathy is well described in volleyball players, whereas axillary neuropathy is usually attributed to dislocation or direct trauma. Concurrent, painless, non-traumatic involvement of both nerves is rare and may be mistaken for disuse or a rotator cuff disorder. **Case Presentation**: A 17-year-old male national-level volleyball player presented with painless atrophy of the dominant shoulder, noticed three months earlier, without trauma, dislocation, instability or pain. Examination showed infraspinatus fossa wasting and predominantly anterior deltoid atrophy, Medical Research Council grade 4 strength in external rotation, forward elevation and abduction, intact lateral deltoid sensation, symmetrical reflexes and a negative Spurling test. Radiographs were unremarkable. Magnetic resonance imaging showed fatty degeneration and atrophy of the infraspinatus and anterior deltoid with relative preservation of the supraspinatus, and no rotator cuff tear, labral tear, paralabral cyst or space-occupying lesion. Motor nerve conduction studies showed a reduced compound muscle action potential recorded from the infraspinatus with a preserved response from the supraspinatus after suprascapular nerve stimulation, together with a reduced deltoid response after axillary nerve stimulation. Selective involvement of the infraspinatus branch was most consistent with a suprascapular lesion at or distal to the branch to the supraspinatus, clinically compatible with involvement around the spinoglenoid notch, and argued against a C5 root or upper trunk lesion. **Conclusions**: Painless shoulder atrophy in an overhead athlete warrants systematic evaluation for peripheral nerve injury. The pattern of motor responses can localize the lesion, but in the absence of needle electromyography a multifocal process such as neuralgic amyotrophy (Parsonage–Turner syndrome) cannot be excluded, and a sport-related traction or compression mechanism should therefore be advanced cautiously.

## 1. Introduction

Peripheral nerve injuries around the shoulder are increasingly recognized in athletes who repeatedly perform overhead motions. The throwing or hitting motion exposes the dominant shoulder to repetitive traction, compression and microtrauma, and the clinical presentation may be subtle when pain is absent [1,2]. Shoulder problems are highly prevalent in this population: more than half of competitive volleyball players report shoulder pain or dysfunction over a season, with the dominant shoulder disproportionately affected [3,4].

In volleyball players, suprascapular neuropathy is one of the best recognized peripheral nerve disorders around the shoulder. It is often attributed to repetitive traction or compression around the spinoglenoid notch and may present as isolated, painless infraspinatus atrophy with weakness of external rotation [5,6,7]. Because range of motion is usually preserved, diagnosis may be delayed until visible atrophy or a decline in performance is recognized.

Axillary neuropathy is less common in athletes and is more often associated with shoulder dislocation, direct trauma or proximal humeral fracture [1,8,9]. Non-traumatic axillary nerve involvement in an overhead athlete is uncommon, and concurrent involvement of the suprascapular and axillary nerves is particularly rare [10,11]. When two nerves that share a C5–C6 origin are affected simultaneously, a single more proximal lesion—cervical radiculopathy, upper trunk or posterior cord brachial plexopathy, or neuralgic amyotrophy—has to be considered before two independent peripheral neuropathies are accepted.

The novelty of the present report should be considered against the existing literature. Isolated, painless suprascapular neuropathy causing infraspinatus atrophy has been described in volleyball players without direct trauma [12], and isolated, painless axillary neuropathy causing deltoid atrophy has similarly been reported in volleyball and beach volleyball players, again without an antecedent traumatic event [13,14]. In the largest available series of suprascapular neuropathy, concomitant axillary neuropathy was identified in 23 of 87 patients, but this combination was more frequently associated with a traumatic mechanism, including motor vehicle accidents and falls, than with a sports-related or idiopathic cause [15]. To our knowledge, painless, non-traumatic, concurrent suprascapular and axillary neuropathy occurring together in a volleyball player, with electrophysiological confirmation of both nerve territories, has not been previously reported; each nerve has been described in isolation in this population, but not the combination (Table 1).

We report a case of painless concurrent partial suprascapular and axillary neuropathy in a young elite volleyball player without trauma, dislocation or shoulder instability, and describe the reasoning by which the lesion was localized, the limits of that reasoning, and the general principles guiding management and return to sport in this setting.

## 2. Case Presentation

### 2.1. History and Physical Examination

A 17-year-old male elite volleyball player, a right-hand-dominant right-wing attacker, presented with visible atrophy of the right shoulder and mild weakness first noticed approximately three months earlier. He denied shoulder pain, anterior shoulder pain, direct trauma, previous shoulder dislocation and symptoms suggestive of recurrent instability. He specifically denied any preceding episode of acute severe shoulder-girdle pain, recent infection, vaccination or unaccustomed exertion, and there was no family history of recurrent neuropathy. He continued training but reported mild weakness during shoulder elevation and hitting.

**Figure 1:**
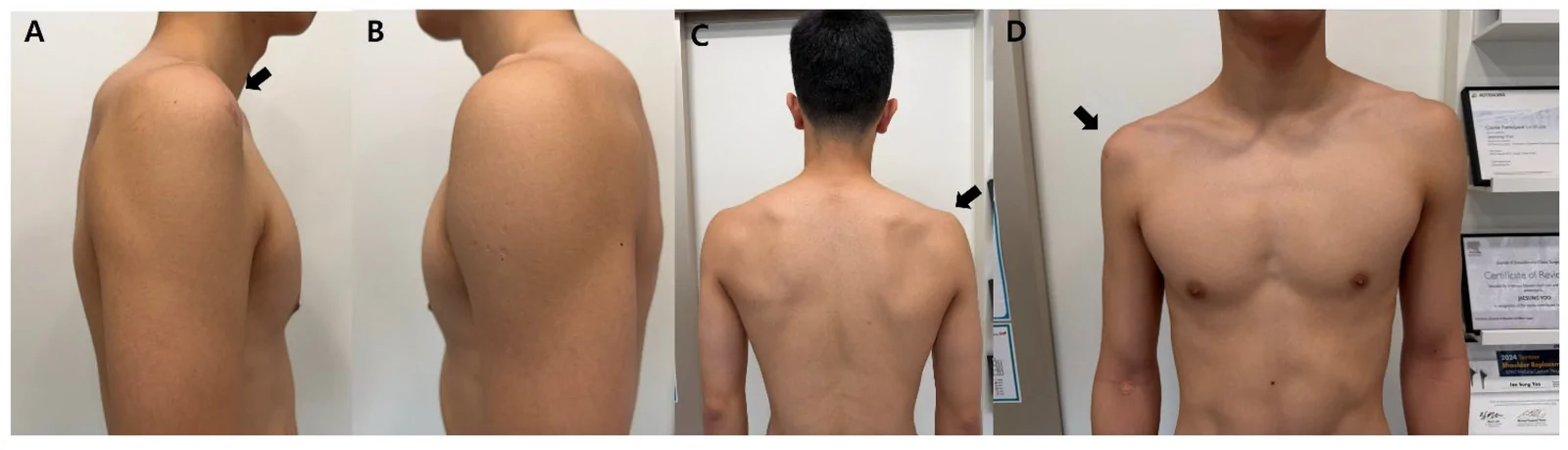
Clinical photographs of the shoulders. (**A**) Lateral view of the affected right shoulder showing flattening and atrophy of the deltoid contour; the black arrow indicates the area of deltoid atrophy. (**B**) Lateral view of the contralateral shoulder for comparison. (**C**) Posterior view demonstrating marked atrophy of the right infraspinatus fossa; the black arrow indicates the depressed infraspinatus fossa. (**D**) Anterior view showing atrophy of the right deltoid muscle, particularly in the anterior portion; the black arrow indicates anterior deltoid atrophy.

On physical examination, active and passive ranges of motion of the right shoulder were full, with no limitation of forward elevation, abduction, external rotation or internal rotation. Inspection revealed marked depression of the right infraspinatus fossa and atrophy of the deltoid, most prominent in its anterior portion. No obvious wasting of the suprascapular fossa was noted clinically; because the supraspinatus lies deep relative to the trapezius, its bulk cannot be assessed reliably by inspection alone, and this clinical impression was therefore interpreted only together with the magnetic resonance imaging (MRI) findings described below (Figure 1).

Manual muscle testing showed Medical Research Council (MRC) grade 4 strength in external rotation, forward elevation and abduction. Elbow flexion, forearm supination, elbow extension, wrist extension and the intrinsic muscles of the hand were graded MRC 5. The external rotation lag sign, Hornblower sign, drop-arm test and Jobe test were negative. Palpable contraction of the deltoid was reduced, but sensation over the lateral deltoid, including the regimental badge area, was intact. Resisted external rotation did not demonstrate additional weakness suggestive of isolated teres minor dysfunction. The Neer and Hawkins impingement signs were negative, and there was no anterior shoulder tenderness or tenderness over the quadrilateral space. The apprehension, relocation and sulcus tests were negative, and no scapular winging was observed. Cervical range of motion was full, the Spurling test did not reproduce radicular symptoms, and the biceps and brachioradialis reflexes were symmetrical.

A concise timeline is as follows: the patient first noticed atrophy approximately three months before presentation; plain radiography, MRI and motor nerve conduction studies were obtained within two weeks of presentation; activity modification together with a scapular and rotator cuff strengthening program was initiated immediately thereafter; and, because of a fixed national-team competition schedule, the patient continued training and competition throughout without interruption. At the time of writing, he reported no shoulder pain or subjective weakness, remained under clinical observation, and continued the strengthening program; formal outcome measures such as repeat imaging, repeat electrodiagnostic testing or quantitative strength testing were not obtained, and the emphasis of this report is accordingly on diagnostic recognition of concurrent nerve involvement rather than on a completed treatment or return-to-sport course.

### 2.2. Imaging

**Figure 2:**
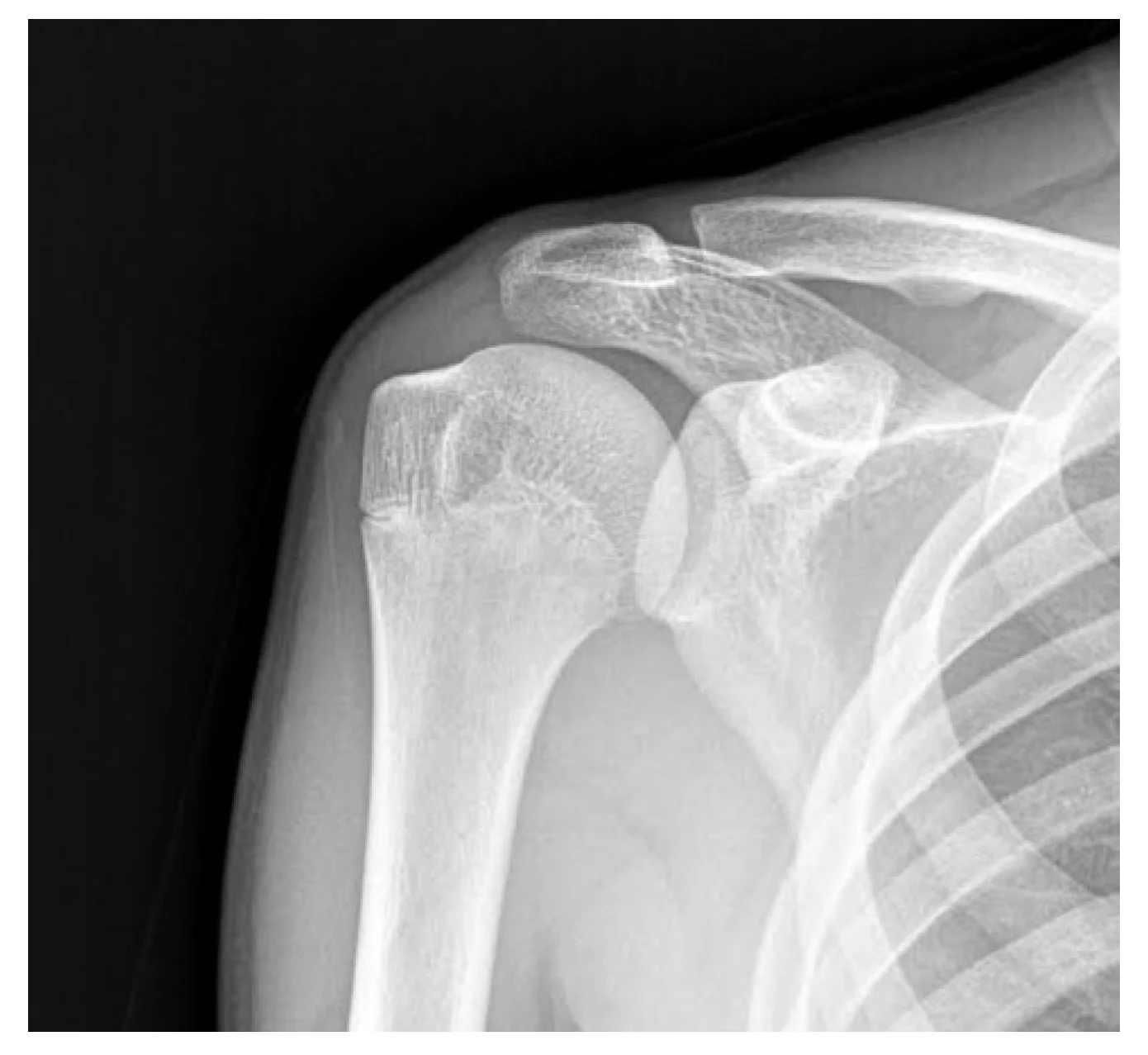
Plain radiograph of the right shoulder. An anteroposterior radiograph showed no osseous abnormality, fracture, dislocation or malalignment.

**Figure 3:**
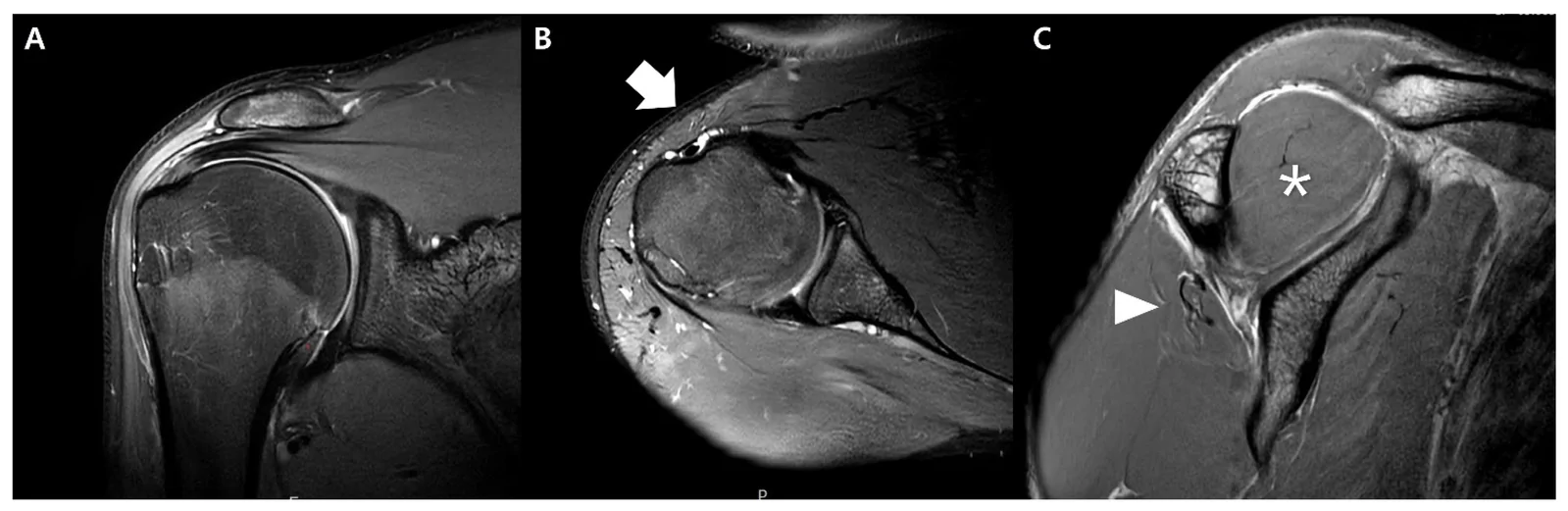
Magnetic resonance imaging findings of the right shoulder. (**A**) Coronal T2-weighted fat-suppressed image showing no definite rotator cuff tear, labral tear, paralabral cyst or other space-occupying lesion. (**B**) Axial T2-weighted fat-suppressed image demonstrating atrophy of the deltoid muscle, particularly in the anterior portion (arrow), with associated signal change and volume loss of the infraspinatus muscle. (**C**) Sagittal T1-weighted image showing fatty degeneration and atrophy of the infraspinatus muscle (arrowhead) with relative preservation of the supraspinatus muscle (asterisk).

Plain radiographs of the right shoulder showed no osseous abnormality, fracture, dislocation or malalignment (Figure 2). MRI demonstrated atrophy and fatty degeneration of the right infraspinatus and atrophy of the deltoid, particularly its anterior portion, with relative preservation of the supraspinatus. There was no evidence of rotator cuff tear, labral tear, paralabral cyst or other space-occupying lesion, and no cyst was identified at the suprascapular or spinoglenoid notch. The teres minor appeared normal, with no signal change or volume loss (Figure 3).

### 2.3. Electrodiagnostic Evaluation

**Figure 4:**
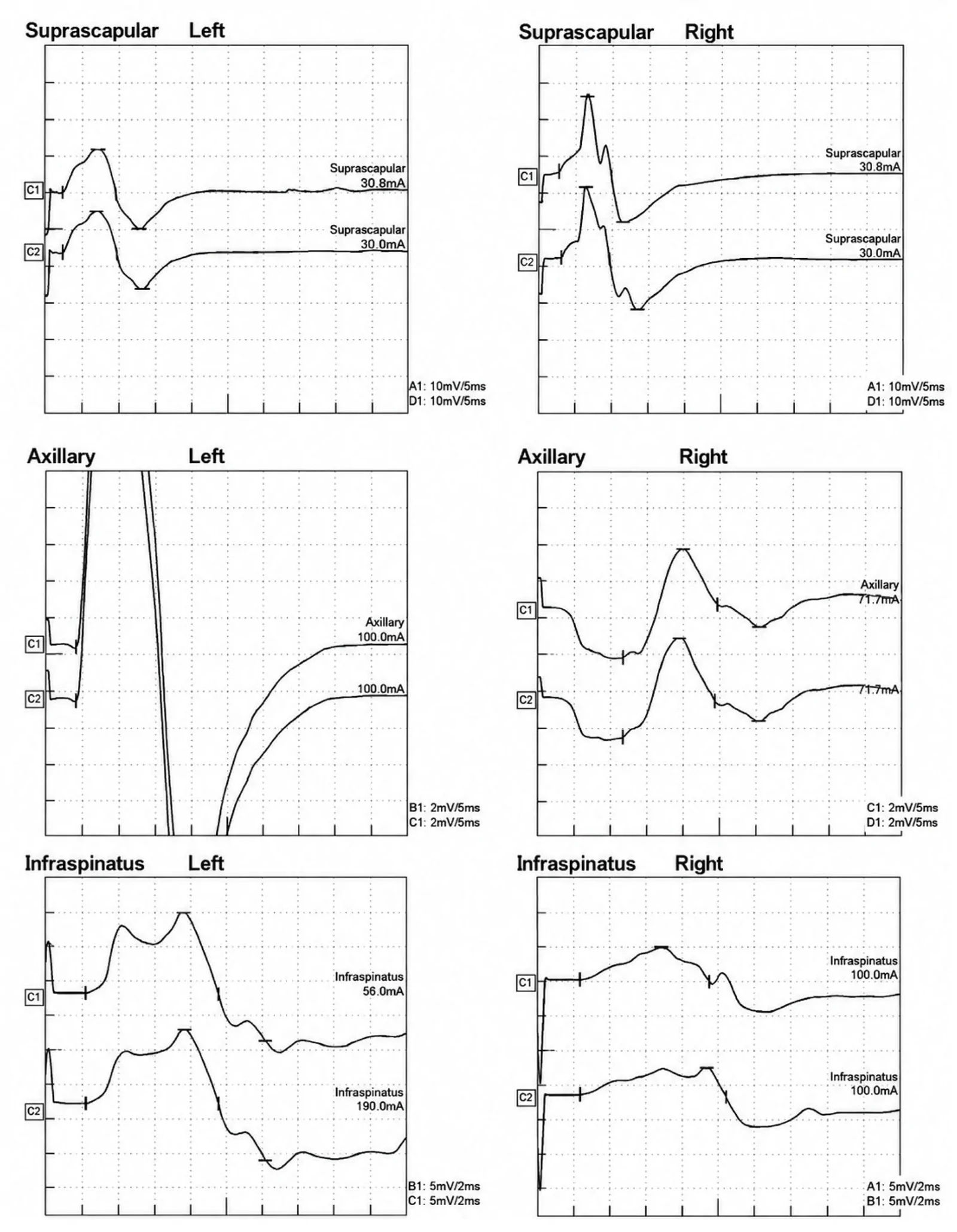
Motor nerve conduction studies of both shoulder girdles, performed at an outside institution and reviewed at our institution from the referring report and accompanying waveform tracings. Each panel shows two superimposed traces recorded at the stimulus intensities indicated, confirming supramaximal stimulation. Upper row: suprascapular nerve stimulation recording from the supraspinatus (**left**, 30.0 and 30.8 mA; **right**, 30.0 and 30.8 mA; gain 10 mV/division, sweep 5 ms/division), showing a preserved and symmetrical response on the affected right side. Middle row: axillary nerve stimulation recording from the deltoid (**left**, 100.0 mA; **right**, 71.7 mA; gain 2 mV/division, sweep 5 ms/division), showing a reduced response on the right. Lower row: suprascapular nerve stimulation recording from the infraspinatus (**left**, 56.0 and 190.0 mA; **right**, 100.0 mA; gain 5 mV/division, sweep 2 ms/division), showing a reduced response on the right. The dissociation between the preserved supraspinatus response and the reduced infraspinatus response is most consistent with a lesion at or distal to the branch to the supraspinatus, clinically compatible with involvement around the spinoglenoid notch. Numerical amplitude and latency values were not independently available beyond the stimulus intensities and traces shown here; see Section 2.3.

Motor nerve conduction studies were performed 2 weeks after presentation at a skin temperature of 36.8 °C at an outside institution; the study was reviewed at our institution from the referring report and the accompanying waveform tracings, and electrode placement, recording technique and the criteria used to determine supramaximal stimulation followed that institution’s standard protocol. Three motor responses were recorded on each side under identical conditions, with the asymptomatic left limb serving as an internal control rather than published normative values, because normative data for these specific recording sites are not well established: the suprascapular nerve recording from the supraspinatus, the axillary nerve recording from the deltoid, and the suprascapular nerve recording from the infraspinatus. Supramaximal stimulation was confirmed at two intensities for each response. The traces are shown in Figure 4; numerical amplitude and latency values from the original report were not available for tabulation.

Two features of this pattern carry the diagnostic weight. First, the dissociation between a reduced infraspinatus response and a preserved supraspinatus response places the suprascapular lesion distal to the origin of the branch to the supraspinatus—that is, at or about the spinoglenoid notch—because a lesion of the C5 root, the upper trunk, or the suprascapular nerve proximal to that branch point would be expected to reduce both responses. Second, the reduced deltoid response indicates concomitant involvement of the axillary nerve, which cannot be attributed to the same suprascapular lesion. The findings were therefore interpreted as partial suprascapular neuropathy at the spinoglenoid notch with concomitant partial axillary neuropathy.

It should be stated explicitly that needle electromyography and sensory conduction studies were not performed. The localization above rests on the pattern of the three motor responses together with the clinical examination, and the implications of this are considered in Section 3.2 and Section 3.5.

### 2.4. Diagnosis and Differential Considerations

On the basis of the clinical presentation, the MRI findings and the motor conduction studies, the patient was considered to have concurrent partial suprascapular and axillary neuropathies in the absence of trauma, shoulder dislocation or clinical instability. The alternative diagnoses considered, and the findings that argued for or against each, are summarized in Table 2.

## 3. Discussion

This case illustrates an uncommon pattern of painless concurrent suprascapular and axillary nerve involvement in a young elite volleyball player. It is clinically instructive because the presenting feature was neither pain, instability nor restricted motion, but visible muscle atrophy with only subtle weakness—a presentation readily misattributed in an overhead athlete to fatigue, disuse or a non-specific decline in performance.

### 3.1. Clinical Recognition

Suprascapular neuropathy has been reported in volleyball players and is commonly associated with infraspinatus atrophy, particularly when the lesion lies at or distal to the spinoglenoid notch [5,6,7]. Its pathophysiology is thought to reflect a combination of nerve traction across the spinoglenoid notch during repetitive overhead motion and, less commonly, direct compression by a paralabral cyst or other space-occupying lesion; recent reviews have clarified this spectrum of contributing mechanisms and the corresponding treatment approaches [16,17,18]. Because glenohumeral motion is preserved and compensatory recruitment of the posterior deltoid and teres minor can maintain functional external rotation, the deficit is often silent until atrophy becomes visible. The posterior shoulder atrophy in this patient was therefore compatible with the pattern described in volleyball players. The more unusual finding was the concomitant deltoid atrophy indicating axillary nerve involvement. Axillary nerve injury in athletes is uncommon and is generally described after traumatic mechanisms such as dislocation or direct impact [1,8,9]; although isolated axillary nerve injury has been reported in athletes, non-traumatic partial axillary neuropathy in a volleyball player is rare [9,10,13,14]. The teres minor forms the superior border of the quadrilateral space through which the axillary nerve travels, and recent anatomical and clinical reviews of quadrilateral space syndrome have emphasized the diagnostic value of assessing this muscle on imaging [19,20]. The absence of dislocation, instability, fracture or a compressive lesion made the present case atypical.

Careful inspection and targeted strength testing were essential. The coexistence of infraspinatus fossa depression with anterior deltoid atrophy indicated that no single structural shoulder lesion could account for the findings, and mild weakness in external rotation, forward elevation and abduction supported a neurogenic origin, while negative lag signs and a negative Jobe test reduced the likelihood of a full-thickness rotator cuff tear. Normal sensation over the regimental badge area did not exclude axillary nerve injury, because partial, motor-predominant involvement is well recognized. It should be emphasized that the apparent sparing of the supraspinatus was established by MRI and by nerve conduction study, not by inspection, since the supraspinatus is covered by the trapezius and its bulk cannot be judged reliably by visual examination alone.

The internal topography of the axillary nerve merits comment. Its anterior branch supplies the anterior and middle deltoid, whereas the posterior branch supplies the posterior deltoid and teres minor and continues as the superior lateral cutaneous nerve of the arm. In this patient, atrophy predominated in the anterior deltoid, sensation over the lateral deltoid was intact, resisted external rotation gave no evidence of teres minor dysfunction, and the teres minor appeared normal on MRI—a constellation compatible with predominant involvement of the anterior branch. This is offered as a possible anatomical explanation rather than a confirmed localization: deltoid bulk was not quantified segmentally, and no needle sampling or sensory conduction study was undertaken to confirm it.

### 3.2. Differential Diagnosis

Because the suprascapular and axillary nerves share a C5–C6 root origin and arise from the upper trunk and the posterior cord, respectively, their simultaneous involvement raises the possibility of a single proximal lesion rather than two independent mononeuropathies. Each principal alternative was therefore examined explicitly (Table 2).

The strongest argument against a proximal lesion in this patient was electrophysiological rather than clinical. The branches to the supraspinatus and to the infraspinatus arise from a common suprascapular nerve, and the branch to the supraspinatus leaves proximal to the spinoglenoid notch. A lesion of the C5 root or of the upper trunk therefore cannot produce a reduced infraspinatus response while leaving the supraspinatus response intact, whereas a lesion at the spinoglenoid notch does precisely that. The observed dissociation, reinforced by the preserved supraspinatus bulk on MRI, by full cervical motion with a negative Spurling test, by the absence of dermatomal sensory loss, by symmetrical biceps and brachioradialis reflexes, and by normal strength of elbow flexion, supination and extension, argues against a C5 radiculopathy, an upper trunk plexopathy and a posterior cord lesion.

These arguments are, however, clinical and inferential rather than definitive. Needle electromyography of the cervical paraspinal, rhomboid, biceps brachii, brachioradialis, triceps brachii and serratus anterior muscles, and sensory conduction studies including the lateral antebrachial cutaneous nerve, were not performed. Subclinical involvement outside the suprascapular and axillary territories, which would have redirected the localization proximally, therefore cannot be formally excluded, and this must temper the confidence attached to the diagnosis.

Neuralgic amyotrophy deserves particular emphasis, since it characteristically produces patchy, multifocal involvement of the shoulder girdle with a predilection for the suprascapular and axillary nerves and a well-described tendency to affect individual branches or fascicles—including, selectively, the branch to the infraspinatus [21,22,23]. The pattern observed in this patient is therefore compatible with neuralgic amyotrophy as well as with a mechanical lesion. The classical phenotype begins with abrupt, severe shoulder-girdle pain that subsides over days to weeks and is followed by weakness and rapid atrophy, frequently after infection, vaccination or unaccustomed exertion; our patient reported no such prodrome and no antecedent illness, and the course was insidious. Nevertheless, painless and forme fruste presentations are described, and neither needle electromyography nor magnetic resonance neurography of the brachial plexus was obtained. Recent series have shown that magnetic resonance neurography can identify hourglass-like fascicular constrictions supporting the diagnosis of neuralgic amyotrophy even when the clinical presentation is atypical [21,22]. Given this branch-level, multifocal distribution, neuralgic amyotrophy should be regarded as a major competing diagnosis in this case rather than a secondary or minor differential, and the current data do not distinguish it from a sports-related mechanical dual mononeuropathy. It is worth noting that needle examination would in any case not have distinguished it decisively, since an inflammatory and a mechanical branch lesion produce identical axonal findings in the same muscles.

Quadrilateral space syndrome would account for axillary nerve involvement alone; it typically produces teres minor denervation, often with posterolateral shoulder pain and quadrilateral space tenderness. Neither was present, and—decisively—an isolated axillary nerve lesion cannot explain the infraspinatus atrophy. A paralabral cyst at the spinoglenoid notch, the most frequent structural cause of suprascapular neuropathy in athletes, was excluded by MRI, as were rotator cuff and labral tears.

### 3.3. Mechanism

The mechanism underlying simultaneous involvement of two nerves in this patient remains uncertain, and we consider it important not to overstate the case for a sport-specific explanation. Volleyball participation in this patient should be regarded as a relevant exposure and clinical context rather than an established cause: repetitive high-level volleyball activity plausibly exposes the shoulder girdle to cumulative traction and compression, and the spinoglenoid notch is an established site at which the infraspinatus branch of the suprascapular nerve may be tethered during the follow-through of the serve and spike [5,6,7,18], but a causal link in this individual patient has not been demonstrated. A mechanical hypothesis for the suprascapular component is therefore reasonable and consistent with the electrophysiological localization to that level, without implying that it has been proven. Extending the same hypothesis to the axillary nerve is less secure: no anatomical site is recognized at which repetitive volleyball activity would predictably injure the axillary nerve in the absence of instability, and this patient had neither dislocation nor apprehension.

Two explanations should therefore be weighed rather than one asserted. The first is that two coincident but independent mechanical mononeuropathies developed in a heavily loaded dominant shoulder; the rarity of this combination in the literature makes coincidence a weak explanation on its own, and it requires an unidentified mechanism for the axillary component. The second is that a multifocal, branch-level neural process—of which neuralgic amyotrophy is the prototype—selectively affected the infraspinatus branch of the suprascapular nerve and the axillary nerve, a distribution highly characteristic of that condition, with the electrophysiological localization reflecting where fascicular damage happened to fall rather than where a compressive force acted. Our data localize the abnormality but do not discriminate between these two mechanisms, because a mechanical and an inflammatory branch lesion produce the same reduction in the same motor responses. In the absence of a cyst, mass, instability or traumatic event, cumulative sports-related microtrauma remains a plausible contributor, but it should be regarded as a hypothesis consistent with the findings rather than as an established mechanism, and the possibility of an inflammatory etiology should be kept in view, particularly in athletes whose deficit does not follow the expected recovery course.

### 3.4. Management and Return to Sport

Non-operative management with activity modification and a scapular and rotator cuff rehabilitation program is the accepted first-line treatment for suprascapular neuropathy in overhead athletes when no compressive lesion is present, with surgical decompression reserved for a structural cause such as a paralabral cyst or for failure of an adequate conservative trial [5,7,11]. The same principle applies to non-traumatic axillary neuropathy without instability [1,9,10]. In this patient the absence of any compressive lesion on MRI, together with preserved range of motion and only grade 4 weakness, favored a conservative approach; a scapular and rotator cuff strengthening program was initiated shortly after diagnosis. This approach is consistent with randomized evidence that structured outpatient rehabilitation targeting scapular control produces measurable benefit in patients with neuralgic amyotrophy and scapular dyskinesia [24]. Because of a fixed national-team competition schedule, activity was not fully interrupted, and the patient continued training and competition throughout; he has remained asymptomatic, without pain or subjective weakness, under continued observation. We emphasize diagnostic recognition rather than a completed management course; the principal message of this report is that painless deltoid and infraspinatus atrophy in an overhead athlete should prompt evaluation for concurrent axillary as well as suprascapular nerve involvement, so that both territories are examined together rather than one being assumed to explain the whole picture.

Two points deserve emphasis for clinicians managing such athletes. Reinnervation after axonal injury proceeds slowly, and visible atrophy and fatty degeneration on MRI may persist long after strength has recovered; imaging is therefore a poor criterion for clearance, and return-to-sport decisions should rest on strength, on sport-specific function and on the absence of compensatory mechanics rather than on the restoration of muscle bulk. In addition, no outcome data exist for concurrent two-nerve involvement of this kind, so the prognosis in this setting is uncertain and counseling should reflect that uncertainty. High-resolution nerve ultrasound is a further diagnostic tool that merits consideration in this setting; it can demonstrate focal nerve enlargement, fascicular abnormalities or focal constrictions, and can therefore complement MRI and electrodiagnostic testing, particularly when neuralgic amyotrophy is part of the differential diagnosis. This is supported by recent literature on ultrasound elastography and imaging of compressed nerves in athletes [25,26,27,28]. Standardized ultrasound protocols for neuralgic amyotrophy and for dynamic compressive neuropathies of the upper extremity in athletes have recently been proposed and may further improve diagnostic yield in comparable cases [25,27].

### 3.5. Limitations

Several limitations should be acknowledged. This is a single case, so no inference about incidence or causation can be drawn. The most important limitation is that the electrodiagnostic evaluation comprised motor conduction studies of the suprascapular and axillary nerves only; needle electromyography was not performed, nor were sensory conduction studies or motor studies of other nerves in the limb. Denervation could therefore not be documented directly, subclinical involvement of muscles outside the two implicated territories could not be sought, and cervical radiculopathy, upper trunk plexopathy and a posterior cord lesion were argued against on the basis of the dissociation between the supraspinatus and infraspinatus responses and of the clinical examination rather than excluded formally. Magnetic resonance neurography of the brachial plexus and high-resolution nerve ultrasound were likewise not performed. As a dynamic, high-resolution technique, ultrasound might have identified focal nerve enlargement, fascicular change, an hourglass constriction or a proximal lesion not evident on shoulder imaging; consequently, neuralgic amyotrophy cannot be excluded [21,25,26]. Strength was graded only by manual muscle testing (MRC scale); handheld dynamometry and side-to-side quantitative strength testing were not performed, so a more granular, quantitative comparison of strength between limbs is not available. The proposed mechanical mechanism is inferential and is not supported by direct anatomical evidence.

## 4. Conclusions

Painless muscle atrophy around the shoulder in an overhead athlete should not be dismissed even when motion is full and pain is absent. Where the pattern of atrophy cannot be explained by a single nerve or tendon lesion, concurrent involvement of more than one peripheral nerve should be considered. In this patient, the dissociation between a reduced infraspinatus response and a preserved supraspinatus response on suprascapular nerve stimulation was most consistent with a lesion at or distal to the branch to the supraspinatus, clinically compatible with involvement around the spinoglenoid notch, and argued against a root or upper trunk lesion, while a reduced deltoid response indicated concomitant axillary nerve involvement. Because needle electromyography was not performed and neuralgic amyotrophy remains unexcluded, the underlying mechanism could not be established, and a sport-related traction or compression mechanism—though plausible—should be advanced cautiously in such cases. We would encourage a complete electrodiagnostic evaluation, including needle examination of muscles outside the suspected territories, whenever multifocal peripheral nerve involvement is suspected in an athlete.

## Figures and Tables

**Table 1:** Comparable published reports of suprascapular and/or axillary neuropathy in volleyball or overhead athletes.

Report	Sport/Population	Nerve(s) Involved	Pain	Mechanism
Tengan et al., 1993 [12]	Volleyball (*n* = 2)	Suprascapular (infraspinatus) only	Painless	Non-traumatic
Paladini et al., 1996 [13]	Volleyball (*n* = 2)	Axillary (deltoid) only	Non-traumatic onset	Non-traumatic
Monteleone et al., 2015 [14]	Beach volleyball (*n* = 2)	Axillary (deltoid) only	Painless (“silent”)	Non-traumatic
Memon et al., 2019 [15]	Mixed clinical series (*n* = 87; 23 with SSN + AN)	Suprascapular ± axillary	Not specified	Predominantly traumatic in the SSN + AN subgroup
Present case	Volleyball (*n* = 1)	Suprascapular + axillary (concurrent)	Painless	Non-traumatic

SSN, suprascapular neuropathy; AN, axillary neuropathy.

**Table 2:** Differential diagnosis of painless concurrent infraspinatus and deltoid atrophy in an overhead athlete.

Differential Diagnosis	Expected Features	Findings in the Present Case
C5 (±C6) radiculopathy	Neck or periscapular pain; C5 dermatomal sensory loss; depressed biceps reflex; positive Spurling test; weakness extending beyond the suprascapular and axillary territories to the biceps brachii and brachioradialis. Because the branches to the supraspinatus and infraspinatus both arise distal to the root, a root lesion would be expected to reduce the supraspinatus response as well as the infraspinatus response.	Full cervical range of motion; negative Spurling test; no radicular pain or dermatomal sensory loss; symmetrical biceps and brachioradialis reflexes; weakness confined to the infraspinatus and deltoid. The supraspinatus response was preserved despite a reduced infraspinatus response. Needle electromyography of the cervical paraspinal muscles was not performed, so a subclinical radiculopathy cannot be formally excluded.
Upper trunk brachial plexopathy	Combined involvement of suprascapular-, musculocutaneous-, axillary- and radial-innervated (C5–C6) muscles, including the supraspinatus, biceps brachii and brachioradialis; sensory abnormality in the lateral antebrachial cutaneous distribution.	Relative preservation of the supraspinatus both on MRI and on nerve conduction study; normal strength of elbow flexion and forearm supination. Needle electromyography and sensory conduction studies were not performed, so a subclinical upper trunk plexopathy cannot be formally excluded.
Posterior cord lesion	Simultaneous axillary and radial nerve involvement, with weakness of elbow and wrist extension in addition to deltoid weakness.	Normal elbow and wrist extension strength. A radial motor conduction study was not performed, so a subclinical posterior cord lesion cannot be formally excluded, although normal strength argues against it clinically.
Parsonage–Turner syndrome (neuralgic amyotrophy)	Abrupt onset of severe shoulder-girdle pain subsiding over days to weeks, followed by patchy, often multifocal weakness and rapid atrophy; predilection for the suprascapular, axillary and long thoracic nerves, frequently at branch or fascicular level; frequent antecedent infection, vaccination or unaccustomed exertion.	No acute painful episode, antecedent illness, vaccination or unaccustomed exertion; symptoms painless throughout with insidious progression over approximately three months; no scapular winging to suggest long thoracic involvement. However, painless and forme fruste presentations are described, and the branch-level, multifocal pattern seen here is characteristic. Neither needle electromyography nor magnetic resonance neurography was performed, and this diagnosis cannot be excluded.
Quadrilateral space syndrome	Isolated axillary nerve compression within the quadrilateral space, typically with teres minor denervation, posterolateral shoulder pain or paraesthesia, and quadrilateral space tenderness.	No posterolateral pain and no quadrilateral space tenderness; no clinical evidence of teres minor dysfunction on resisted external rotation testing, and the teres minor appeared normal on MRI. Decisively, an isolated axillary nerve lesion cannot account for the infraspinatus involvement.
Spinoglenoid or suprascapular notch cyst or mass	Paralabral cyst or other space-occupying lesion compressing the suprascapular nerve, frequently with an associated labral tear.	MRI demonstrated no paralabral cyst, labral tear, or other space-occupying lesion at either notch.
Rotator cuff tear or tendinopathy	Pain, positive lag signs, weakness on provocative testing, structural tendon defect on MRI.	Negative external rotation lag, Hornblower, drop-arm and Jobe tests; MRI showed an intact rotator cuff.

MRI, magnetic resonance imaging.

## Data Availability

The data presented in this study are contained within the article. Further enquiries may be directed to the corresponding author. Restrictions apply to the availability of the clinical record because of patient privacy.

## Abbreviations

The following abbreviations are used in this manuscript:

MRC	Medical Research Council
MRI	magnetic resonance imaging

## References

[1] Safran M.R. (2004). Nerve injury about the shoulder in athletes, part 1: Suprascapular nerve and axillary nerve. Am. J. Sports Med..

[2] Bowers R.L., Cherian C., Zaremski J.L. (2022). A review of upper extremity peripheral nerve injuries in throwing athletes. PM&R.

[3] Skazalski C., Whiteley R., Sattler T., Kozamernik T., Bahr R. (2024). Knee, low back, and shoulder problems among university and professional volleyball players: Playing with pain. J. Athl. Train..

[4] Keller R.A., De Giacomo A.F., Neumann J.A., Limpisvasti O., Tibone J.E. (2018). Risk factors for shoulder injuries in female athletes playing overhead sports: A systematic review. Sports Health.

[5] Witvrouw E., Cools A., Lysens R., Cambier D., Vanderstraeten G., Victor J., Bellemans J. (2000). Suprascapular neuropathy in volleyball players. Br. J. Sports Med..

[6] Holzgraefe M., Kukowski B., Eggert S. (1994). Prevalence of latent and manifest suprascapular neuropathy in high-performance volleyball players. Br. J. Sports Med..

[7] Mazza D., Iorio R., Drogo P., Gaj E., Viglietta E., Rossi G., Fedeli G., Ferretti A. (2021). Did the prevalence of suprascapular neuropathy in professional volleyball players decrease with the changes occurred in serving technique?. Phys. Sportsmed..

[8] Visser C.P.J., Coene L.N.J.E.M., Brand R., Tavy D.L.J. (1999). The incidence of nerve injury in anterior dislocation of the shoulder and its influence on functional recovery: A prospective clinical and EMG study. J. Bone Jt. Surg. Br..

[9] Probst D.T., Mackinnon S.E., Prather H. (2019). Isolated axillary nerve injury in an elite high school American football player: A case report. Sports Health.

[10] Lee S., Saetia K., Saha S., Kline D.G., Kim D.H. (2011). Axillary nerve injury associated with sports. Neurosurg. Focus.

[11] Ayik G., Kolac U.C., Kaymakoglu M., McFarland E., Huri G. (2025). Dark side of the shoulder: Suprascapular and axillary nerve compressions. Int. Orthop..

[12] Tengan C.H., Oliveira A.S.B., Kiymoto B.H., Morita M.P.A., Medeiros J.L.A., Gabbai A.A. (1993). Isolated and painless infraspinatus atrophy in top-level volleyball players: Report of two cases and review of the literature. Arq. Neuropsiquiatr..

[13] Paladini D., Dellantonio R., Cinti A., Angeleri F. (1996). Axillary neuropathy in volleyball players: Report of two cases and literature review. J. Neurol. Neurosurg. Psychiatry.

[14] Monteleone G., Gismant M., Stevanato G., Tiloca A. (2015). Silent deltoid atrophy in beach volleyball players: A report of two cases and literature review. Int. J. Sports Phys. Ther..

[15] Memon A.B., Dymm B., Ahmad B.K., Sripathi N., Schultz L., Chandok A. (2019). Suprascapular neuropathy: A review of 87 cases. Muscle Nerve.

[16] Strauss E.J., Kingery M.T., Klein D., Manjunath A.K. (2020). The evaluation and management of suprascapular neuropathy. J. Am. Acad. Orthop. Surg..

[17] Bozzi F., Alabau-Rodriguez S., Barrera-Ochoa S., Ateschrang A., Schreiner A.J., Monllau García J.C., Perelli S. (2020). Suprascapular neuropathy around the shoulder: A current concept review. J. Clin. Med..

[18] Le Hanneur M., Maldonado A.A., Howe B.M., Mauermann M.L., Spinner R.J. (2019). “Isolated” suprascapular neuropathy: Compression, traction, or inflammation?. Neurosurgery.

[19] Hangge P.T., Breen I., Albadawi H., Knuttinen M.G., Naidu S.G., Oklu R. (2018). Quadrilateral space syndrome: Diagnosis and clinical management. J. Clin. Med..

[20] Dalagiannis N., Tranovich M., Ebraheim N. (2020). Teres minor and quadrilateral space syndrome: A review. J. Orthop..

[21] Gstoettner C., Mayer J.A., Rassam S., Hruby L.A., Salminger S., Sturma A., Aman M., Harhaus L., Platzgummer H., Aszmann O.C. (2020). Neuralgic amyotrophy: A paradigm shift in diagnosis and treatment. J. Neurol. Neurosurg. Psychiatry.

[22] Gabet J.M., Anderson N., Groothuis J.T., Zeldin E.R., Norbury J.W., Jack A.S., Jacques L., Sneag D.B., Poncelet A. (2025). Neuralgic amyotrophy: An update in evaluation, diagnosis, and treatment approaches. Muscle Nerve.

[23] van Eijk J.J.J., Groothuis J.T., van Alfen N. (2016). Neuralgic amyotrophy: An update on diagnosis, pathophysiology, and treatment. Muscle Nerve.

[24] Janssen R.M., Lustenhouwer R., Cup E.H., van Alfen N., Ijspeert J., Helmich R.C., Cameron I.G.M., Geurts A.C.H., van Engelen B.G.M., Graff M.J.L. (2023). Effectiveness of an outpatient rehabilitation programme in patients with neuralgic amyotrophy and scapular dyskinesia: A randomised controlled trial. J. Neurol. Neurosurg. Psychiatry.

[25] Cignetti N.E., Cox R.S., Baute V., McGhee M., van Alfen N., Strakowski J.A., Boon A.J., Norbury J.W., Cartwright M.S. (2023). A standardized ultrasound approach in neuralgic amyotrophy. Muscle Nerve.

[26] Dąbrowska-Thing A., Zakrzewski J., Nowak O., Nitek Ż. (2019). Ultrasound elastography as a potential method to evaluate entrapment neuropathies in elite athletes: A mini-review. Pol. J. Radiol..

[27] Nguyen C.T., Chou R.C. (2025). Diagnostic ultrasonography of upper extremity dynamic compressive neuropathies in athletes: A narrative review. Int. Orthop..

[28] Bordalo M., Gulde M.L.S., Hagert E. (2025). Imaging on the painful and compressed nerve: Upper extremity. Int. Orthop..

